# Height-to-Diameter Ratio and Porosity Strongly Influence Bulk Compressive Mechanical Properties of 3D-Printed Polymer Scaffolds

**DOI:** 10.3390/polym14225017

**Published:** 2022-11-18

**Authors:** José I. Contreras Raggio, Carlos Toro Arancibia, Carola Millán, Heidi-Lynn Ploeg, Ameet Aiyangar, Juan F. Vivanco

**Affiliations:** 1 Facultad de Ingeniería y Ciencias, Universidad Adolfo Ibáñez, Viña del Mar 2580335, Chile; 2 Swiss Federal Laboratories for Materials Science and Technology (EMPA), 8600 Dübendorf, Switzerland; 3 Facultad de Artes Liberales, Universidad Adolfo Ibáñez, Viña del Mar 2580335, Chile; 4 Department of Mechanical and Materials Engineering, Queen’s University, Kingston, ON K7L3N6, Canada

**Keywords:** polymer scaffolds, 3D printing, height:diameter ratio, porosity, pore size, mechanical properties

## Abstract

Although the architectural design parameters of 3D-printed polymer-based scaffolds—porosity, height-to-diameter (H/D) ratio and pore size—are significant determinants of their mechanical integrity, their impact has not been explicitly discussed when reporting bulk mechanical properties. Controlled architectures were designed by systematically varying porosity (30–75%, H/D ratio (0.5–2.0) and pore size (0.25–1.0 mm) and fabricated using fused filament fabrication technique. The influence of the three parameters on compressive mechanical properties—apparent elastic modulus E_app_, bulk yield stress σ_y_ and yield strain ε_y_—were investigated through a multiple linear regression analysis. H/D ratio and porosity exhibited strong influence on the mechanical behavior, resulting in variations in mean E_app_ of 60% and 95%, respectively. σ_y_ was comparatively less sensitive to H/D ratio over the range investigated in this study, with 15% variation in mean values. In contrast, porosity resulted in almost 100% variation in mean σ_y_ values. Pore size was not a significant factor for mechanical behavior, although it is a critical factor in the biological behavior of the scaffolds. Quantifying the influence of porosity, H/D ratio and pore size on bench-top tested bulk mechanical properties can help optimize the development of bone scaffolds from a biomechanical perspective.

## 1. Introduction

Porous scaffolds to guide and stimulate tissue growth are increasingly considered a viable option in bone tissue engineering applications. Optimal osteogenic signal expression and subsequent differentiation of cells seeded on the scaffold are influenced by physical scaffold parameters such as mean porosity, pore size and pore interconnectivity and mechanical parameters such as strength and elastic modulus of the fabricated bulk structure [1,2,3,4]. Porosity and interconnectivity ensure migration, attachment proliferation and differentiation of cells in the scaffold and flow for nutrient transport and waste evacuation [5]. Similarly, scaffold macro-pore size is an important variable affecting the ability of bone scaffolds to accommodate cell ingrowth and new bone formation [5,6,7,8]. Although an ideal scaffold pore size for efficient bone regeneration has yet to be determined, studies have reported viable pore sizes ranging from 100 μm up to 1200 μm [7,9,10,11,12,13,14,15]. 

Different types of scaffold architectures have been implemented over the last several decades, which can be classified according to their macro-porous configuration: single random porous domain, single regular porous domain and multi-domain porous [16]. The main limitation of single random porous domain scaffolds, for example sponge-type scaffolds, is that seeded cells cannot migrate into the interior regions of the scaffold. Additive manufacturing (AM), also known as rapid prototyping, has emerged as a powerful technique to address the limitation of single random porous domain scaffolds by creating scaffolds with a single macropore domain of regular morphology, such as orthogonal arrays of channels [17]. Hence, the lack of inter-connectivity presented in random pore structures is removed and flow of nutrients through the internal architecture can be facilitated. Another advantage of AM, in contrast to conventional and subtractive fabrication, is the fabrication of tissues, organs and medical devices with complex shapes and multiple materials [18]. Fused filament fabrication (FFF), which is based on heating thermoplastic filaments to their fusion point in order to fabricate a structure in a layer-by-layer process, is a popular AM method [19]. The resolution of FFF theoretically supports a minimum feature size of 100 μm [20]. In addition, FFF is generally inexpensive and therefore, together with the described advantages, the most commonly used polymer-based three-dimensional (3D) printing method for bone tissue scaffolds.

From a mechanical perspective, the bone scaffold structure should have sufficient mechanical strength to withstand normal physiological loading during the bone regeneration phase [21,22,23]. Furthermore, the stiffness of the scaffold must be tuned according to the mechanical properties of the surrounding tissue—i.e., to enable load-sharing conditions for optimal bone growth without overloading the nascent bone. This macro-mechanical requirement is typically assessed by conducting quasi-static compression tests on fabricated test specimens to determine elastic modulus (measure of bulk stiffness) and yield stress (bulk mechanical strength). Specimens used for compression testing are fabricated with the same architectural design parameters of porosity and pore size as the bone scaffold. However, these mechanical test specimen requirements give rise to several issues.

Firstly, the strength and stiffness are often bulk values, i.e., they are based on an assessment of bulk stress computed as overall applied compressive force over bulk cross-sectional area. The bulk cross-sectional area is based on the overall specimen footprint and typically does not account for the internal porous structure of the specimen, which significantly alters the *effective* cross-sectional area. Consequently, the elastic modulus is an *apparent* elastic modulus and can vary depending on the designed porosity or pore size. For 3D printed scaffolds, there is additional variability across specimens fabricated to achieve the same designed porosity and pore size due to the limitations of precision of the 3D printing process.

Secondly, the accuracy of compression testing results for trabecular bone and biomimetic cellular solid structures is strongly affected by the presence of end-artifacts [24]. End-artifacts can broadly be classified into two categories: specimen-platen interface conditions and structural end-artifacts [25]. End-artifacts distort results more strongly in shorter specimens compared to taller specimens. To standardize the mechanical characterization of porous scaffolds, international standards of traditional polymer based-materials have been widely adopted by several research groups [26,27,28]. For instance, the American Society for the Testing of Materials’ (ASTM) ASTM D695 standard for compressive properties of rigid plastics defines the standard test specimen for strength measurements to be in the form of a prism or cylinder whose aspect-ratio, defined as height/diameter (H/D), is a minimum of 2/1 [29]. Nevertheless, scaffolds studies often report compression test results with lower H/D ratios—as low as 0.15 [30,31,32,33,34]. Although this H/D ratio may be sufficient to meet minimum requirements for the continuum assumption and is adequate for biological experiments to assess cell toxicity, proliferation and adhesion, it may result in an inaccurate characterization of mechanical property. 

While studies have recognized these issues on a qualitative basis, their impact, especially from a biomechanical perspective, has neither been thoroughly quantified nor explicitly discussed when reporting mechanical properties of bone scaffolds. Hence, a better quantitative understanding and awareness of the influence of porosity, pore size and H/D-ratio on bone scaffold mechanical properties is needed to optimize the development of these scaffolds for tissue engineering. Therefore, in this study, controlled bone scaffold architectures were designed by systematically varying three parameters: porosity, H/D ratio and pore size, and 3D printed with FFF. The influence of the three parameters and their interactions on scaffold mechanical properties such as Elastic modulus, Yield stress and Yield strain were investigated through a multiple linear regression adjustment by a stepwise multiple linear regression model.

## 2. Materials and Methods

Porous mechanical test specimens were fabricated with a commercial 3D printer in deliberate combinations of pore size, porosity and H/D ratio. To assess the impact of the parameters on the mechanical properties—apparent elastic modulus, yield stress and yield strain—a stepwise multiple linear regression model-based study was conducted.

### 2.1. Material and 3D Printing of Scaffold Test Specimens

Mechanical test specimens were fabricated with commercially acquired polylactic acid (PLA) 1.75 mm diameter filament using a desktop FFF 3D printer (Mbot Grid II+, Hangzhou, China) at 210 °C and 60 mm/s printing speed [35]. The printing parameters are listed in Table 1.

### 2.2. Scaffold Test Specimen Design and Fabrication Process

**Figure 1:**
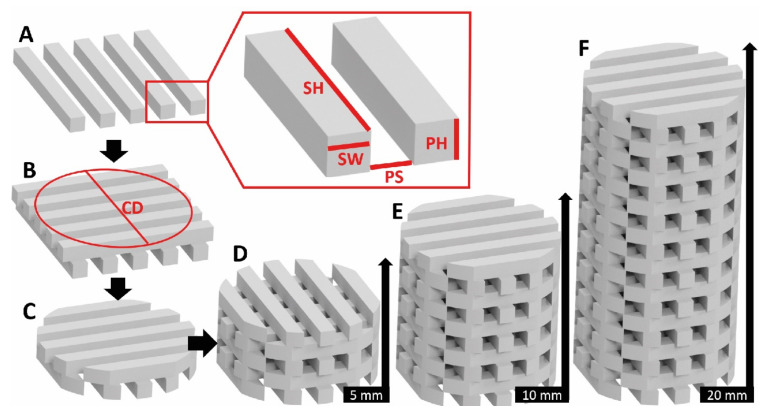
Scaffold test specimen geometrical design process to match theoretical values of porosity, heights and pore size: (**A**) the first layer was designed, the region of interest (ROI) of the struts that are created with a width (SW), length (SH) and a height (PH), as denoted by red lines. PS corresponds to the pore size and is equal to the pore height (PH). (**B**) A second layer is added by rotating the first one by 90° and placing it on top of it, a circumference with diameter (CD) is designed and everything outside it is removed producing (**C**). (**D**–**F**) The remained part is duplicated along the cylindrical principal axis (*z*-axis) as required for the specimen height. The final specimen geometry with length of 5, 10 or 20 mm was exported for 3D printing as a STL file.

Mechanical test geometries were based on common scaffold designs to compare with published studies and followed the ASTM D695 standard for mechanical characterization of polymers. The mechanical testing specimens were cylindrical with a constant diameter, D, of 10 mm. Inner architectures were designed following the procedure delineated in Figure 1 by varying three main parameters: (1) Height (H/D ratio), (2) Porosity and (3) Pore size. Each parameter had three levels: low, medium and high, as explained below:

1. Height, H: A “low” height value of 5 mm represented a 1/2 H/D ratio (D = 10 mm) and is commonly used in biological assessment. End-effects, as defined by St. Venant’s principle, tend to be significant in these geometries. To minimize influence of end-effects, ASTM D695 defines an H/D ratio of 2/1. Accordingly, a ”high” height value of 20 mm was defined in this study. To effectively compare the mechanical behavior and the influence of the end effects, a “medium” height value of 10 mm was additionally defined representing an H/D ratio of 1/1. Thus, the respective H/D ratios were 0.5, 1.0 and 2.0.
2. Porosity: Scaffold designs generally mimic the porosity of bone tissue. Low porosity structures such as cortical bone range between 5–30% porosity, while cancellous bone porosity is mostly in the range of 75–95% [36]. In this study, a “low” porosity level close to 30% and a “high” level near 75% were defined. The “medium” porosity was 50%.
3. Pore size: In the current study, pore sizes from 0.25 to 0.5 mm were defined as the “low” level. Pores from 0.5 mm until 0.75 mm were “medium” level and pore sizes from 0.75 to 1.00 mm were the “high” level.

**Figure 2:**
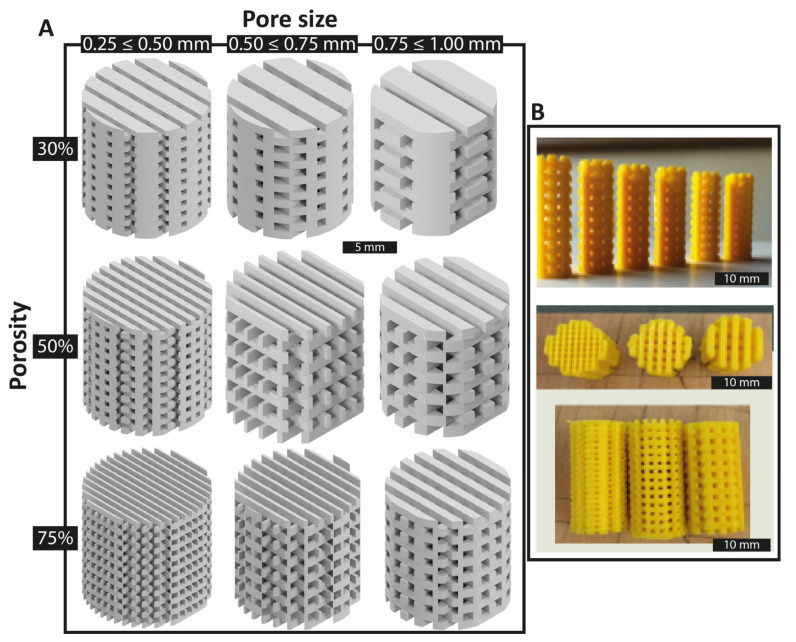
Scaffold test specimen geometries: specimens with different inner architectures were created due to the combinations between pore size and porosity. (**A**) Representative specimen design of 10 mm height with a H/D ratio of 1.0, is shown for the different combinations of porosity and pore size. (**B**) Actual printed samples based on (**A**).

Figure 2 summarizes the different combinations of pore size and porosity for a representative specimen height of 10 mm. The designed scaffolds were printed for each condition in the horizontal printing plane, where orientation of the fibers and their bonding was enhanced over other planes [37]. Table 2 summarizes the experimental design with the scaffold design combinations. Based on the combination of parameters—six specimens each with three levels for each of the three factors (6 × 3^3^)—a total of 162 specimens were fabricated.

### 2.3. Morphology Characterization

Diameter and height of each printed specimen were recorded as a mean of three measurements for each dimension, as measured with a set of calipers. Scaffold porosity was measured with a buoyancy scale following the Archimedes method [38]. In this case ethanol, with density of 0.789 g/mL was used as the liquid with known density at room temperature of 22 °C. Porosity was calculated based on the formula:

Porosity = 1 − {(W_d_ − W_s_)/(V × ρ)} (1)

where: W_d_ is the dry weight measured before the immersion; W_s_ the submerged weight acquired in the balance; V the overall volume; and ρ the porosity of the displaced liquid. 

The porosity, as calculated based on Equation (1), was the experimentally measured porosity of the 3D-printed specimens. The theoretical, design porosity—i.e., either 30%, 50% or 75%—was confirmed based on the CAD model as the effective volume of the scaffold material (total volume of the struts) divided by the bulk volume (*H* × *π* × *D*/4) of the cylinder. Differences between the experimentally measured porosity and the design porosity were then expressed as percentages.

**Figure 3:**
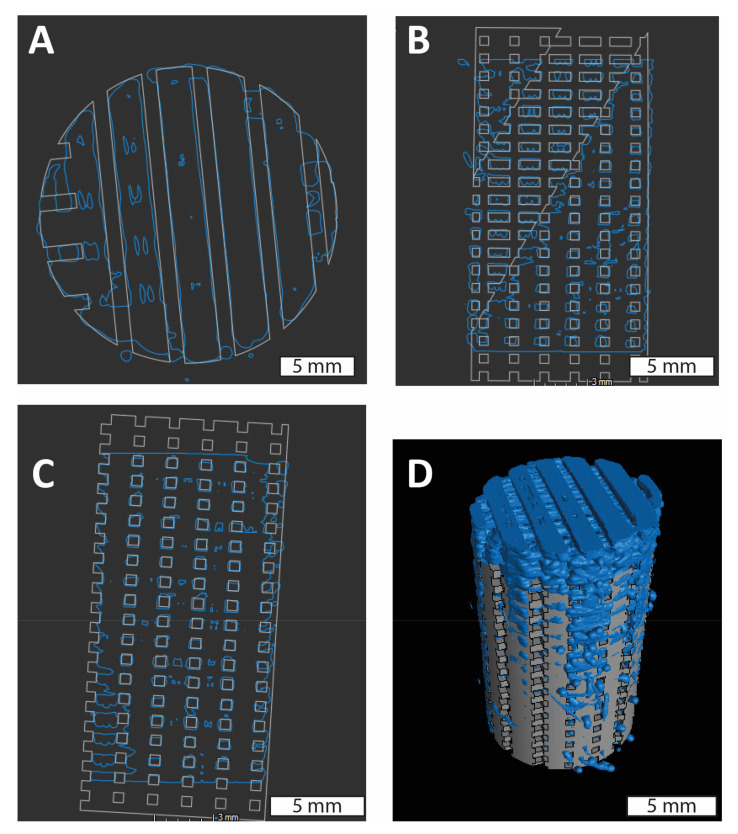
Qualitative evaluation of the accuracy of a printed specimen geometry versus theoretical CAD design: A comparison between the CAD model (grey) and the acquired volume with micro CT data (blue). (**A**) A cross-sectional plane at the midpoint along the horizontal plane showing the inner correlation; (**B**) along central vertical plane; (**C**) along the vertical plane with a small angle of rotation; and, (**D**) Isometric view of micro CT data (blue) and CAD model (grey) overlayed.

Following porosity measurements, specimens were dried and stored for subsequent evaluations. Optical measurements were performed to measure the specimen pore size with digital pictures acquired by an optical microscope (Leica, Leica Camera AG, Wetzlar, Germany). Furthermore, micro computed tomography (micro-CT) scans were conducted to verify the inner structure of the samples (Figure 3). Images were acquired in an EasyTom micro (Rx Solutions, Boynton Beach, FL, USA) using a configuration of voltage of 90 kV and current of 200 μA, frame rate of 2 fps. Each scan of 360° took 20 min to achieve a resolution of 10 μm. First, the software X-Act (Rx Solutions, Boynton Beach, FL, USA) was used to preprocess the images to generate a dataset of layers along the z axis of the scan volume. These images were analyzed in VG Studio and compared with the designed CAD for printed irregularities. The samples were imaged by placing them in a low-density material to avoid undesired rotation of the specimen while scanning.

### 2.4. Scaffold Test Specimen Mechanical Property Characterization

Compression tests were performed at room temperature on a universal materials testing machine (Test Resources, Shakopee, MN, USA) at a fixed, quasi-static speed of 1.27 mm/min following the standard ASTM D695 [39]. Specimens were placed in the center of the plate and a preload of 50 N was applied. The test was conducted until specimen nominal strain was at least 30% strain. Bulk stress and strain were computed as:

(2) $$\mathrm{Bulk}\;\mathrm{stress}:\;\sigma =\frac{F}{CSA_{bulk}};\;\mathrm{bulk}\;\mathrm{strain}:\;\varepsilon =\frac{\delta }{L_{o}}$$

where: *F* = force applied at the crosshead; *CSA_bulk_* = nominal cross-sectional area; δ = crosshead displacement; *L_o_* = initial length (height) of the specimen.

Hooke’s Law of elasticity in elastic solids was applied to calculate apparent elastic modulus as follows: *σ* = E_app_×ε; where σ is the bulk compressive stress, E_app_ the apparent elastic modulus and ε the bulk strain. Apparent elastic modulus, E_app_, was found by linear regression of stress–strain data from the linear segment of the test data, generally between 0 and 2% strain. The Yield stress and Yield strain were determined with a 1% offset strain [40].

### 2.5. Data Analysis

Statistical analysis was performed using R (version 3.6.3) [41]. Results were expressed as means and standard deviations and, in all cases, the level of significance was set at α = 0.05. First, Spearman’s coefficients [42] were calculated to determine the correlation between the response variables and the possible explanatory variables (Height, Porosity and Pore Size). Next, effects of Height, Porosity and Pore Size were assessed based on a Mann-Whitney-Wilcoxon Test to identify statistically significant effects on the response variables. Based on the results obtained in the Mann-Whitney-Wilcoxon Test for each explanatory variable, a multiple linear regression model was developed by using the measured experimental values of the explanatory variables and their respective interactions. The linear model was:

Y = (Height) × β_1_ + (Porosity) × β_2_ + (Pore size) × β_3_ + (Height:Porosity) × β_4_ + (Porosity:Pore size) × β_5_ + Intercept (3)

where Y are the response variables, namely, E_app_, Yield Stress and Yield Strain. β_i_ are the coefficients of the regression model associated with the variable i, namely, Height, Porosity, Pore size and their respective interactions.

Subsequently, in order to determine which variables contributed to the multiple linear regression model, a step-wise regression algorithm by the forward method [43] was applied to define the influence of the independent variables. Normality assumptions inherent to the multiple linear regression model were verified with the Kolmogorov-Smirnov test [44].

## 3. Results

### 3.1. Scaffold Test Specimen Morphology Characterization

The 3D printed scaffold test specimens had consistent and uniform bulk dimensions (height and diameter), with low standard deviations and errors. The variation in height (H) across the three H/D ratio groups was ≤2%, while the variation in diameter (D) was less than 6%. Given the low variation in the bulk dimensions, H/D ratio was maintained as a categorical variable with three levels (H/D = 0.5, 1.0 and 2.0) for the statistical analysis. The variability between design and printed structures was verified by superimposing the micro CT-based volume of the printed scaffold onto the CAD model. (Figure 3).

In contrast, variation in the internal architecture of the printed samples, represented by porosity and pore size, was much larger than was found for the bulk dimensions. For example, compared to low and medium porosity, specimens with high porosity (75%) had a larger error, with 10–50% percent errors between the measured and theoretical porosity. The medium porosity (50%) samples had smaller errors, 11–21%. Samples with low porosity (30%) generally had the lowest error, 0–8%, except for an atypical error of 27% for the medium pore size sub-group (0.75 mm). The “low” pore size sub-group (0.50 mm) had the biggest percent error of 46–50%, followed by the “medium” size (0.75 mm) with 19–20% and the “large” size (1 mm), 10–11%. As a result, porosity and pore size were treated as continuous variables in subsequent statistical analyses.

### 3.2. Scaffold Test Specimen Mechanical Property Characterization

**Figure 4:**
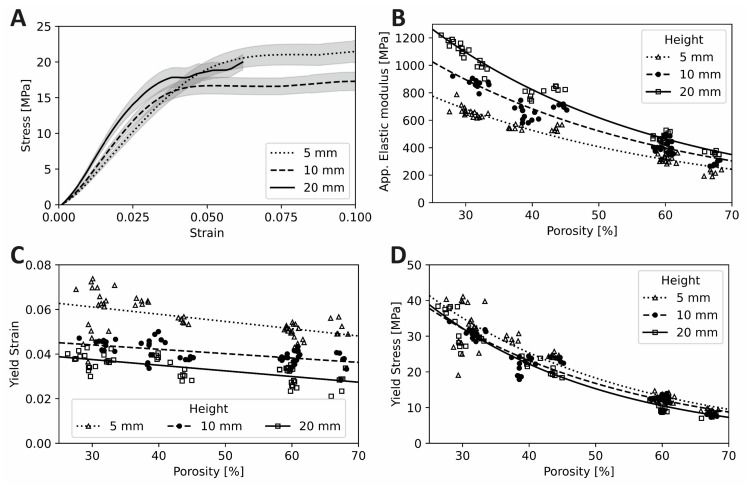
Mechanical properties of the scaffold test specimens: (**A**) Stress–strain curves showing the average and standard error for all the samples grouped by height. (**B**) Elastic modulus versus porosity for three specimen heights. (**C**) Yield strain versus porosity for three specimen heights. (**D**) Yield stress versus porosity for three specimen heights. Grey, shaded bands in (**A**) represent the standard error.

Apparent elastic modulus, E_app_, was positively correlated with specimen H/D ratio and 20 mm height specimens (largest H/D ratio = 2.0) had, on average, the largest E_app_, which decreased progressively for the 10 mm and 5 mm height groups. Yield strain, on the other hand, exhibited a strong negative correlation. Specimen H/D ratio did not influence the bulk Yield stress (Table 3, Figure 4D).

Pore size, as an independent variable, did not have a significant effect on E_app_ of the specimens in this configuration; however, porosity did have an effect. At the highest levels of porosity, E_app_ decreased, as expected with porous structures. Yield stress values also decreased with increase in porosity, while E_app_ and yield stress were strongly negatively correlated with porosity. Yield strain exhibited a mild negative correlation (Figure 4C). 

**Figure 5:**
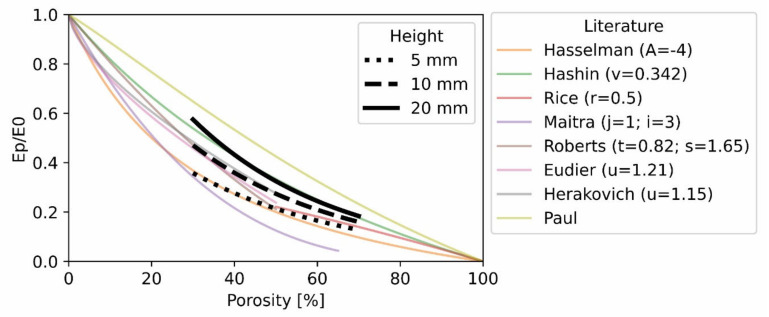
Standardized modulus (measured elastic Modulus E_p_ divided by the material modulus E_0_) versus the porosity for three specimen heights. Data from the current study is overlayed with published curves [45,46,47,48,49,50,51,52].

Apparent elastic modulus and yield stress were related to porosity with an exponential decay, ae^bx^ (Figure 4B,D, respectively). Yield strain was linearly related to porosity (Figure 4C). The normalized modulus (apparent elastic modulus divided by the material elastic modulus versus porosity curves) are overlayed with published curves in Figure 5 according to ASTM standard D696. A summary of the measurements from the morphology and mechanical property characterization can be found in Table 3.

### 3.3. Multiple Linear Regression Analysis

Statistical analyses were performed to evaluate the correlation between the different parameters. Spearman correlation was used to obtain the nonparametric measure of rank correlation. This correlation describes how well the relationship can be defined using a monotonic function. Table 4 shows Spearman correlation coefficients between the independent variables (Height, Porosity and Pore Size) and the response variables (E_app_, yield stress and yield strain). Out of the three response variables, height exhibited the highest correlation with yield strain (−0.761) followed by E_app_ (0.501) and negligible correlation with yield stress (0.043). Porosity was highly correlated with both E_app_ (−0.859) and yield Stress (−0.912), but had a low correlation with yield strain (−0.269). Finally, pore size was not strongly correlated with any of the three response variables, the highest coefficient being −0.204 for yield stress. 

Table 5 shows the step-wise statistical analysis results, with models for the response variables (E_app_, yield stress and yield strain) based on the specimen parameters (height, porosity and pore size). The model successfully explained up to 96% of the variation in both apparent elastic modulus (E_app_*)* and yield stress. For E_app,_ porosity was the principal parameter (R^2^ = 73%), followed by height (R^2^ = 19%). For yield stress, the principal parameter was porosity (R^2^ = 94%), which explained almost all the variation in yield stress. Finally, the model explained only up to 72% of the variation in yield strain, which was mainly represented by the height (R^2^ = 60%), with porosity accounting for the remaining 12%.

## 4. Discussion

One hundred and sixty-two 3D-printed scaffold test specimens with controlled geometries were fabricated to systematically evaluate the variation in the mechanical response of 3D structures obtained based on variations in H/D ratio, porosity and macro-pore size. Combined, these parameters resulted in almost a six-fold variation in the full range of apparent elastic modulus and bulk yield stress values– from 189 MPa to 1220 MPa and from 7 MPa to 41 MPa, respectively.

Results from the statistical analysis can help us understand how the parameters tested in this study affect mechanical properties.

### 4.1. Elastic Modulus

In Table 5, the E_app_ is well represented with the proposed model (R^2^ of 96%) with porosity as the principal influencing parameter (R^2^ of 73%), similar to findings in literature [18,53,54,55,56,57]. The negative β_i_ suggests that the increase of porosity reduced the stiffness of the samples with a *β_i_* of −8.92 per percentage increase in porosity. 

H/D ratio had a relatively smaller influence on the E_app_ (R^2^ of 19%) within the model, but a higher sensitivity on the samples with a value of β_i_ of 43.46. Moreover, the model also revealed an interaction or mild confounding effect between the height and porosity. For example, specimens with high porosity (negative influence on E_app_), but high H/D ratio (positive effect on E_app_) exhibited elastic modulus values close to specimens with low porosity and low H/D ratio (Figure 4B). A sensitivity study was carried out on the intercept value of the model to extrapolate the response of a solid sample. The model, driven by height with null porosity and thus null pore size, showed an E_app_ of 1034, 1251 and 1684 [MPa]—a variation of almost 60%—purely due to changes in specimen height between 5 mm (H/D = 0.5), 10 mm (H/D = 1.0) and 20 mm (H/D = 2.0), respectively. These values are consistent with those reported previously with the same configuration [20], which serves as a further validation of the model. The change in *E_app_* for each different height essentially represents the effect of the H/D ratio in the mechanical response [35,58]. Thus, although the influence of the specimen H/D ratio on the elastic modulus relative to specimen porosity may be smaller, it is still significant and must be taken into account while comparing across studies. 

Finally, neither the range of pore sizes nor its interaction with the other variables (height and porosity) had a significant effect on E_app_ (*p* > 0.05), which is consistent with literature [18,54,55,56,57,59]. 

### 4.2. Yield Strain

Compared to elastic modulus the relative influence of H/D ratio and porosity on yield strain were more or less reversed. The regression model had a lower predictive strength, predicting variation in the yield strain with an R^2^ of 72%. H/D ratio was the principal influencing parameter (R^2^ of 60%). The influence of porosity was relatively smaller (R^2^ of 12%). Although the influence of these parameters on yield strain has been noted in past studies [35,58], this study quantifies these effects in a model where the sensitivity is minimum for both parameters, with β_i_= -0.0014 for height and β_i_= -0.000276 for porosity. Hence, for a given material, neither the inner architecture, nor the H/D ratio changes affected the yield strain substantially, with a consistent value of 0.07. Furthermore, the pore size did not have a significant influence on the mechanical responses of the proposed model, as has also been shown in the literature [3]. 

### 4.3. Yield Stress

Finally, similar to apparent elastic modulus, yield stress and its variations are also represented well by the model (R^2^ = 96%). However, porosity variations could explain most of the variations in yield stress (R^2^ of 94%) and with a low representation by the height (R^2^ of 2%). The *β_i_* shows that yield stress was inversely correlated with these parameters, a trend consistent with previous studies [18,55,56,57,59]. Notably, however, yield stress was the only response variable, where pore size displayed a significant influence (*p* < 0.05); nevertheless, the influence within the model was low (R^2^ < 1%). 

**Figure 6:**
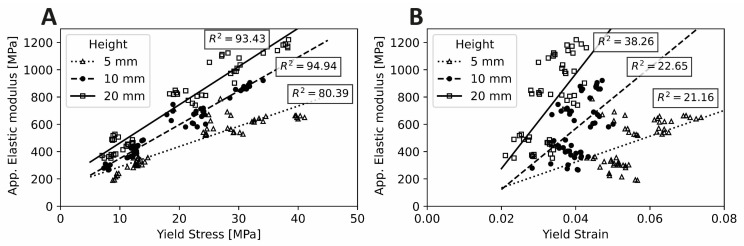
Correlation between mechanical properties of the scaffold test specimens: (**A**) Yield Stress with respect to Apparent Elastic modulus and (**B**) Yield Strain with respect to Apparent Elastic modulus. Lines represent a linear fit with R^2^ (%) being the coefficient of determination.

**Figure 7:**
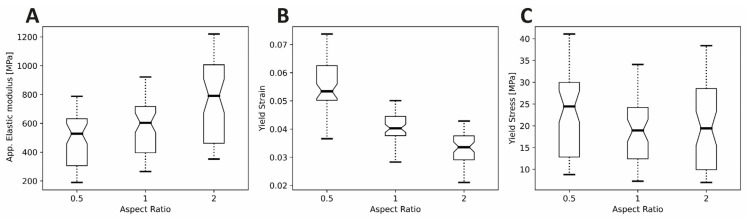
Mechanical analysis of the scaffold test specimens with respect to the H/D ratio: (**A**) Apparent Elastic modulus, (**B**) Yield Strain and (**C**) Yield Stress. Data are presented as notched box plots. Boxes represent the second and third quartile around the median, which is represented by the thick horizontal line within the block. Whiskers represent 100% of the data within each group, including outliers. Notches represent a 95% confidence interval (CI_notch_) of the median and extend to [±1.58 × IQR/((*n*)^0.5^)]. IQR = interquartile range between first to third quartile and “*n*” = number of non-missing observations within the group. Non-overlapping notches represent significant differences [60,61].

The yield stress findings can be understood as essentially a product of E_app_ and yield strain. Further, E_app_ is strongly correlated with yield stress, while it is poorly correlated with yield strain (Figure 6). Both E_app_ and yield strain are strongly negatively influenced by porosity, resulting in an extremely strong negative effect of porosity on yield stress. On the other hand, while E_app_ was positively affected by specimen H/D ratio (Figure 7A), yield strain was negatively affected (Figure 7B), essentially cancelling out the effect of specimen height on the yield stress (Figure 7C). Consequently, yield stress appeared less sensitive to variations in specimen H/D ratio.

## 5. Conclusions

Within the context of the three factors investigated together in the current study, H/D ratio and porosity of the fabricated structures had a strong influence on the mechanical properties commonly used to understand the mechanical behavior of these structures, namely apparent elastic modulus, yield strain and yield stress. Thus, when comparing across studies, for example, between printing techniques or even choosing candidate polymer materials for printing scaffolds, it is important to note the differences in the H/D ratios as well as porosities of the specimens used in the respective studies. Particularly for porosity, the variations in actual porosity of the fabricated specimens with respect to the designed value may also be significant enough to influence the reported mechanical properties. Depending on the specific mechanical property in consideration, either porosity or H/D ratio may have a dominating influence on the results; nevertheless, variations in both should be taken into account. Although H/D ratio appeared to significantly influence the stiffness (elastic modulus) and yield strain, yield stress did not seem sensitive to this factor within the specific range of H/D ratios investigated in this study. Thus, yield stress could potentially be a benchmark mechanical property for comparisons across studies for specimens with different heights, as the height does not have a high effect on yield stress. On the other hand, if the samples have different porosity but the same height, the yield strain is a suitable result variable for comparisons. 

## Figures and Tables

**Table 1:** Fabrication parameters for 3D printing of scaffold test specimens.

Extrusion temperature (°C)	210
Bed temperature (°C)	24
Nozzle diameter (mm)	0.4
Layer thickness (mm)	0.15
Extrusion speed (mm/s)	60
Travel speed (mm/s)	90
Printing direction (°)	0 and 90

**Table 2:** Scaffold test specimen geometries, constant diameter of *D* = 10 (mm).

Parameters	Levels:	Low	Medium	High
Porosity (%)		~30	~50	~70
Height (mm)		5	10	20
Pore size (mm)		0.25–0.50	0.50–0.75	0.75–1.00
H/D ratio		0.5	1.0	2.0

**Table 3:** Design parameters and specimen measured morphology and mechanical properties. Designed pore sizes were Low (L): 0.25–0.50 mm, Medium (M): 0.5–0.75 mm and High (H): 0.75–1.00 mm. Actual pore size was considered as a continuous variable in the statistical analysis. Apparent elastic modulus (E_app_), yield stress (σ) and yield strain (ε_y_) were determined from compression test data. Measurements from 3D printed scaffold test specimens were based on six replicates and results are expressed as mean ± standard deviation.

Design Parameters			Measured Morphology and Mechanical Properties (*n* = 6)			
Height	Porosity	Pore size	Porosity (%)	E_app_ (MPa)	*σ*_y_ (MPa)	ε_y_ (%)
5	30	L–M–H	30.57 ± 1.40	659.77 ± 39.25	32.93 ± 6.25	6.03 ± 1.08
	50	L–M–H	54.69 ± 7.84	393.48 ± 106.94	16.74 ± 5.62	5.10 ± 0.41
	75	L–M–H	54.82 ± 12.6	361.03 ± 139.91	17.35 ± 8.66	5.57 ± 0.60
10	30	L–M–H	33.80 ± 3.38	802.87 ± 99.06	27.39 ± 5.40	4.26 ± 0.41
	50	L–M–H	54.53 ± 7.22	515.64 ± 134.45	16.33 ± 5.09	3.76 ± 0.14
	75	L–M–H	55.97 ± 11.9	423.73 ± 143.24	14.31 ± 6.48	4.15 ± 0.52
20	30	L–M–H	29.94 ± 2.04	1086.85 ± 85.69	31.95 ± 4.49	3.74 ± 0.35
	50	L–M–H	54.31 ± 7.43	586.79 ± 159.47	13.19 ± 4.30	2.96 ± 0.34
	75	L–M–H	55.67 ± 11.5	522.31 ± 187.85	13.79 ± 6.60	3.29 ± 0.56

**Table 4:** Spearman correlation coefficients between the study variables.

	Independent Variables			Response Variables		
	Height	Porosity	Pore Size	E_app_	Yield Stress	Yield Strain
Height	1	−0.113	0.009	0.501	−0.043	−0.761
Porosity	-	1	0.255	−0.859	−0.912	−0.269
Pore size	-	-	1	−0.178	−0.204	−0.135
E_app_	-	-	-	1	0.784	−0.112
Yield Stress	-	-	-	-	1	0.481
Yield Strain	-	-	-	-	-	1

**Table 5:** Step-wise statistical analysis with models for the three response variables. Coefficient of determination (R^2^), coefficient of the regression model associated with the variable (β_i_) and p-value (*p*-value) are listed for each of the variables and their interactions, were *p*-value < 0.0001: ****; *p*-value < 0.001: ***; *p*-value < 0.01: **; *p*-value < 0.05: *; *p*-value >= 0.05: NA (does not contribute to the model).

Y	X	$R^{2}$	$\beta_{i}$	*p*-Value	
E_app_ (MPa)	Intercept		817.31	<0.0001	****
	Height	0.19	43.36	<0.0001	****
	Porosity	0.73	−8.92	<0.0001	****
	Pore size	NA	NA	NA	
	Height:Porosity	0.04	−8.92	<0.0001	****
	Porosity:Pore size	NA	NA	NA	
Final Model:	E_app_ = (Height) × β_1_ + (Porosity) × β_2_ + (Height:Porosity) × β_4_ + Intercept				
Yield Stress (MPa)	Intercept		52.93	<0.0001	****
	Height	0.02	−0.19	<0.0001	****
	Porosity	0.94	−0.67	<0.0001	****
	Pore size	0.003	2.49	0.002	**
	Height:Porosity	NA	NA	NA	
	Porosity:Pore size	NA	NA	NA	
Final Model:	Yield stress = (Height) × β_1_ + (Porosity) × β_2_ + (Pore size) × β_3_ + Intercept				
Yield Strain	Intercept		0.07	<0.0001	****
	Height	0.60	−1.40 × 10^−3^	<0.0001	****
	Porosity	0.12	−2.76 × 10^−4^	<0.0001	****
	Pore size	NA	NA	NA	
	Height:Porosity	NA	NA	NA	
	Porosity:Pore size	NA	NA	NA	
Final Model:	Yield strain = (Height) × β_1_ + (Porosity) × β_2_ + Intercept				

## Data Availability

The data presented in this study can be made available on request from the corresponding author.
